# Determinants of health insurance ownership among South African women

**DOI:** 10.1186/1472-6963-5-17

**Published:** 2005-02-28

**Authors:** Joses M Kirigia, Luis G Sambo, Benjamin Nganda, Germano M Mwabu, Rufaro Chatora, Takondwa Mwase

**Affiliations:** 1 World Health Organization, Regional Office for Africa, Brazzaville, Congo; 2 Department of Economics, University of Nairobi, Nairobi, Kenya

## Abstract

**Background:**

Studies conducted in developed countries using economic models show that individual- and household- level variables are important determinants of health insurance ownership. There is however a dearth of such studies in sub-Saharan Africa. The objective of this study was to examine the relationship between health insurance ownership and the demographic, economic and educational characteristics of South African women.

**Methods:**

The analysis was based on data from a cross-sectional national household sample derived from the South African Health Inequalities Survey (SANHIS). The study subjects consisted of 3,489 women, aged between 16 and 64 years. It was a non-interventional, qualitative response econometric study. The outcome measure was the probability of a respondent's ownership of a health insurance policy.

**Results:**

The χ^2 ^test for goodness of fit indicated satisfactory prediction of the estimated logit model. The coefficients of the covariates for area of residence, income, education, environment rating, age, smoking and marital status were positive, and all statistically significant at p ≤ 0.05. Women who had standard 10 education and above (secondary), high incomes and lived in affluent provinces and permanent accommodations, had a higher likelihood of being insured.

**Conclusion:**

Poverty reduction programmes aimed at increasing women's incomes in poor provinces; improving living environment (e.g. potable water supplies, sanitation, electricity and housing) for women in urban informal settlements; enhancing women's access to education; reducing unemployment among women; and increasing effective coverage of family planning services, will empower South African women to reach a higher standard of living and in doing so increase their economic access to health insurance policies and the associated health services.

## Background

A health system in any country performs instrumental functions of stewardship (oversight), creation of resources (investment and training), delivering services (provision), and financing (collecting, pooling and purchasing) [1]. Ultimately, the effectiveness and efficiency with which these functions are executed determine the extent to which a health system achieves its intrinsic goals of improving health, responding to people's non-medical expectations, and fairness of financial contributions. For instance, the extent to which South Africa's post-apartheid government will be able to attain its vision of creating a caring and humane society in which all citizens have access to affordable good quality health care will depend on the performance of its national health system [2].

Prior to the 1994 democratic elections in South Africa, the health system was built on the apartheid ideology, which was characterized by racially segregated health services, geographical disparities, fragmentation, duplication and specialized hospital-centred services favouring the urban populations [3]. The health system's functions were inefficiently and inequitably performed. The health system was not effective in: improving the health status of the majority of the formerly disenfranchised South Africans; responding to their legitimate non-medical expectations; and ensuring their social protection from the impoverishing catastrophic health expenditures [4]. Consequently, provision of primary health services was for a long time neglected and inequitable [34]. The worsening poverty situation in some provinces has made the primary health services inaccessible to the majority of the population [35].

In an attempt to overcome the above-mentioned public health challenges, the post-apartheid democratic governments have introduced several reforms in the health sector: creation of a quasi-federal structure with one national and nine provincial departments of health (provincial legislatures and bureaucracies); establishment of a district health system, including expansion and upgrading of the primary care infrastructure; and health care financing [5]. The latter included: use of a population and need-based resource allocation mechanism by the National Department of Health up to 1995 (after which each province receives a block grant directly from the national treasury); the removal of public sector fees for pregnant and lactating women, children under six years of age and all those who use the public primary health care system; and the enactment of a Medical Insurance Schemes Act aimed at regulating the medical insurance schemes industry more effectively.

In spite of the abovementioned policy interventions, primary health care still remains under funded. For example, in 2003/2004 financial year, South Africa spent a total of 36.9 billion Rands (1 Rand = US$6) on health: 61.3% was spent on hospitals curative care, 16.1% on primary health care, 1.8% on HIV/AIDS treatment, 2.3% on nutrition, 4.0% on emergency services, 4.3% on administration, 2.5% on training, 1.6% on support services and 6.1% on other services [34].

According to Doherty and McLeod [6] and McIntyre et al. [7], the primary objectives of the Medical Insurance Schemes Act of 1998 are to: (i) increase the number of people covered; (ii) improve health-related cross subsidization within individual medical insurance schemes and curtail exclusion of high-risk groups, e.g. the elderly; (iii) prevent 'dumping' of medical insurance scheme members on public hospitals by requiring the schemes to cover a prescribed minimum package of hospital services for all members; and (iv) ensure effective health care cost containment.

In 2001, there were 146 registered medical schemes (i.e. those falling fully under the Act) and eight Bargaining Council schemes (i.e. those granted exemption from certain provisions of the Act), all covering a total of 7 million people [6], i.e. less than 20% of the South African population. An additional 2 million people were covered by private insurance or industry-specific health services [7]. Unfortunately, there has been no significant increase in the size of the population covered by the medical schemes since the implementation of the Act. For instance, in 2003 South Africa had uninsured population of 38.6 million people: 15.1% were from Eastern Cape province, 6.0% from Free State, 17.8% from Gauteng, 22.1% from Kwazulu Natal, 12.8% from Limpopo, 7.2% from Mpumalanga, 1.7% from Northern Cape, 8.5% from North West and 8.7% from Western Cape [34].

The objective of this study was to examine the relationship between individuals' demographic, economic and educational characteristics, and their likelihood of being insured.

## Methods

### Conceptual framework

There are two kinds of risks involved in health care: (i) the risk of becoming ill, with the accompanying loss in the quality of life, cost of medical care, loss of productive time during illness and, in more serious cases, death; and (ii) the risk of total or incomplete or delayed recovery [8].

Welfare economics of uncertainty predicts that individuals would like to insure against both forms of risks. The theory of expected utility, on which this study is based, assumes that each individual strives to maximize the expected value of a utility function; individuals are normally risk-averse, meaning that they have a diminishing marginal utility of income; and health risks for different individuals are basically independent, so that pooling them reduces the risk to the insurer to relatively small proportions [9].

In the South African National Health Inequalities Survey (SANHIS) [10], the respondents were asked the following question: "Does anyone in this household belong to a medical aid or health insurance scheme? 1 = Yes, 2 = No". Given the dichotomous nature of this question, we shall assume that the potential health insurance consumer faces the choice between purchase of some or no insurance. The consumer chooses between the two prospects on the basis of the utility expected from each.

The potential consumers of insurance are assumed to make decisions based on the magnitude of the perceived difference between the level of expected utility with insurance (EU_1_) and expected utility without insurance (EU_2_). We need to analyse the effect of changes in the independent variables on the difference in the level of the expected utility of the two prospects, i.e. EU_1 _minus EU_2_. If the difference were equal to zero, the consumer would be expected to be indifferent between the two prospects. However, if the difference were greater than 0, then the risk-averse consumer would be expected to opt for insurance [11].

The empirical model used in the analysis of individual household's choice between having no health insurance and having health insurance is presented in the *Appendix*. The variables included in the model are defined in Table 1.

**Table 1:** Definition of variables

***Variable***	***Variable description***
Health insurance ownership	1 = if the respondent has health insurance; 0 otherwise
Health rating	1 = if self-evaluated health status is excellent, very good or good; 0 otherwise
Environment rating	1 = if the respondent feels that the environment she lives in is good, very good or excellent; 0 otherwise
Residence	1 = if the respondent resides in either metro formal area, metro transitional area, smaller city/town formal area, smaller city/town transitional area, or rural white farms; 0 = metro informal area, smaller city/town informal area, or rural – "homeland"
Income	Total monthly gross income in Rand (US$≈6 Rand)
Education	Respondent's education level: 1 = matriculation (standard 10 or secondary school) and above; 0 = below matriculation
Age	Respondent's age in years
Age squared	Respondent's age squared
Race	1 = if respondent is white; 0 if person of colour
Household size	Total number of persons in a household
Occupation	1 = if a white-collar worker; 0 otherwise
Employment status	1 = if unemployed and looking; 0 = otherwise
Smoking	1 = if the respondent smokes cigarettes; 0 otherwise
Alcohol use	1 = if the respondent drinks alcohol; 0 otherwise
Contraceptive use	1 = if respondent uses a contraceptive; 0 = otherwise
Marital status	1 = if married; 0 = single, separated or divorced

Since economic theory does not provide much guidance on model specification, the choice of explanatory variables in the current study were guided by the past health insurance demand studies undertaken in the U.S.A. [12-15], Europe [16-18] and Israel [19,20].

Once again, based on past health insurance choice analysis studies, the coefficients of the variables included in equation 2 in the *Appendix *would, a priori be expected to assume the signs indicated in Table 2.

**Table 2:** Hypothesized relationships between the dependent variable (insurance ownership) and independent variables

***Independent Variables***	***Variable coefficient***	**Expected sign**	**Studies from which the hypothesized signs are based**
Health rating	*B*_1_	Negative	Trujillo [23], Coasta and Garcia [28]
Environment rating	*B*_2_	Indeterminate	
Residence	*β*_3_	Positive	Liu and Chen [31]
Income	*β*_4_	Positive	Deb et al [22], Trujillo [23], Vera-Hernandez [26], Coasta and Garcia [28], Besley et al [32]
Education	*β*_5_	Positive	Deb et al [22], Trujillo [23], Vera-Hernandez [26], Coasta and Garcia [28], Besley et al [32], Liu and Chen [31]
Age	*β*_6_	Positive	Trujillo [23], Liu and Chen [31], Grossman [12]
Age squared	*β*_7_	Negative	Trujillo [23], Grossman [12]
Race	*β*_8_	Indeterminate	
Household size	*β*_9_	Negative	Deb et al [22], Vera-Hernandez [26], Besley et al [32]
Occupation	*β*_10_	Positive	Vera-Hernandez [26]
Employment status	*β*_11_	Negative	Vera-Hernandez [26], Liu and Chen [31]
Smoking	*β*_12_	Indeterminate	
Alcohol use	*β*_13_	Indeterminate	
Contraceptive use	*β*_14_	Indeterminate	
Marital status	*β*_15_	Positive	Rhine et al [21], Trujillo [23], Liu and Chen [31]

A Chi-Squared test (χ^2^) for independence was undertaken to test the relationship between health insurance ownership and the individual independent variables. The null hypothesis is that the two variables are independent of one another (because there is a significantly large difference between the observed data and the calculated expected data). The alternate hypothesis is that the two variables are dependent on each other (because there is not a significant difference). Thus, for health rating, the hypotheses are as follows: (i) H_0_: A person's health insurance ownership status and their health rating are independent or unrelated; and (ii) H_A_: A person's health insurance ownership status and their health rating are related (dependent on each other).

### Data

The data for empirical analysis were taken from the 1994 South African National Health Inequalities Survey, a household survey of a randomly selected sample of the South African population between the ages of 16 and 64 years [10]. The full sample was 3,796 persons, out of which 3,489 were women. Our analysis focused on the latter. The data set was rich with economic, demographic, social and health characteristics of the respondents.

## Results

### Descriptive statistics

Table 3 presents the frequency and percentage distribution of the dependent and independent variables. Overall, 30% of the women in the sample said that they had a household member with a health insurance policy. Ninety five percent of the respondents who reported to have got a household member belonging to a medical aid or health insurance scheme lived in formal city dwellings and/or on farms owned by white South Africans.

**Table 3:** Frequencies and percentages for explanatory variables

**Variables**	**With insurance: frequency (%)[N = 1044)**	**Without insurance: frequency (%) [N = 2445]**	**Chi-square (p-value)**
Health rating: 1 = Excellent/very good/good	435 (41.67)	1238 (50.63)	23.57 (*p *< 0.0001)
0 = Fair and poor	609 (58.33)	1207 (49.37)	
Environment rating: 1 = Good/very good/Excellent living environment	491 (47.03)	1580 (64.62)	93.843959 (P < 0.0001)
0 = Fair or poor	553 (52.97)	865 (35.38)	
Residence: 1 = Formal city dwellings + white farms	995 (95.31)	1625 (66.46)	325.45 (P < 0.0001)
0 = Informal dwellings +"former homelands"	49 (4.69)	820 (33.54)	
Income (in Rand): No regular income	137 (13.12)	275 (11.25)	1238.93 (P < 0.0001)
1 – 950	100 (9.58)	1497 (61.23)	
951 – 1900	179 (17.15)	435 (17.79)	
1901 – 3800	317 (30.36)	191 (7.81)	
3801 – 7600	223 (21.36)	38 (1.55)	
7600 +	88 (8.43)	9 (0.37)	
Education: 1 = Matriculation (standard 10) and above	566 (54.21)	2205 (90.18)	579.15 (P < 0.0001)
0 = Below matriculation	478 (45.79)	240 (9.82)	
Age (in years): 16 – 25	128 (12.26)	328 (13.42)	16.53 (P = 0.0024)
26 – 35	299 (28.64)	666 (27.24)	
36 – 45	284 (27.20)	586 (23.97)	
46 – 55	189 (18.10)	404 (16.52)	
56 and above	143 (13.70)	461 (18.85)	
Race: 1 = African, Coloured & Indian	947 (90.71)	1981 (81.02)	50.87 (P < 0.0001)
0 = White	97 (9.29)	464 (18.98)	
Household size: 1 – 4 household members	647 (61.97)	1043 (42.66)	123.30 (P < 0.0001)
5 – 8	351 (33.62)	1114 (45.56)	
9 – 12	41 (3.93)	240 (9.82)	
13 and above	5 (0.48)	48 (1.96)	
Occupation: 0 = White-collar worker	326 (31.23)	168 (6.87)	357.05 (P < 0.0001)
1 = Blue-collar worker	718 (68.77)	2277 (93.13)	
Employment status: 1 = Involuntarily unemployed	967 (92.62)	1933 (79.06)	95.94 (P < 0.0001)
0 = Voluntarily unemployed or employed	77 (7.38)	512 (20.94)	
Smoking: 1 = If a cigarette smoker	292 (27.97)	607 (24.83)	3.78 (P = 0.0519)
0 = Not a cigarette smoker	752 (72.03)	1838 (75.17)	
Alcohol use: 1 = Alcohol drinker	148 (14.18)	276 (11.29)	5.71 (P = 0.0168)
0 = Not alcohol drinker	896 (85.82)	2169 (88.71)	
Contraceptive use: 0 = Uses contraceptives	213 (20.40)	399 (16.32)	8.43 (P = 0.0037)
1 = Does not use contraceptives	831 (79.60)	2046 (83.68)	
Marital status: 1 = Married	627 (60.06)	1144 (46.79)	51.53 (P < 0.0001)
0 = Single, separated, divorced	417 (39.94)	1301 (53.21)	

In the overall sample, the median income was 849.5 Rand and the mean was 1546.4 Rand, with a standard deviation (STD) of 1998.2 Rand. The sub-sample of the households without health insurance had an average income of 853 Rand (STD = 1073) compared to 3172 Rand (STD = 2623) in the group with insurance. Seventy-seven percent of the households with at least one member with health insurance had a monthly income of more than 951 South African Rand, compared to only 28% among the group without health insurance.

The average and median household size in the overall sample was five members, with a standard deviation of about 3. The group with health insurance had an average household size of four people (STD = 2.1) compared to 5 people (STD = 2.7) in the group without insurance. Fifty-seven percent of the sub-sample without health insurance had a household size of more than five members compared to 38% among the group with health insurance.

The group with health insurance had an average age of 40 years (STD = 12.5) compared to 41 years (STD = 14.1) in the group without insurance. The average age in the whole sample was 40.9 years (STD = 13.6) and the median was 39 years.

As alluded to in the methods section, a Chi-Squared test for independence was undertaken to test the relationship between health insurance ownership and the individual independent variables. The results are presented in the last column of Table 3. Since the computed Chi-Squared values for health rating, environment rating, residence, income, education, age, race, household size, occupation, employment status, alcohol use, contraceptive use and marital status were greater than their respective critical Chi-Squared values (at 5% significance level) we reject the null hypotheses and conclude that the row and column variables in Table 3 are not independent. Thus, for example in the case of health rating, we would accept the alternative hypothesis (H_A_) that a person's health insurance ownership status and their health rating are related (dependent on each other).

### Regression analysis

Table 4 provides odds ratios, 'p' values, coefficients, and 't' test values. The t-test is used to test the hypothesis (i.e. H_0_: β = 0) about individual regression slope coefficients. The 't' values for individual variables are obtained by dividing their coefficients (e.g. β_INCOME_) by their standard errors (e.g. SE_INCOME_). For example, the coefficient for income is 0.0004727 and the standard error is 0.0000339, and given H_0_: β = 0, the relevant t-value is indeed 13.946 as specified in Table 4:

**Table 4:** Logistic model regression results

**Explanatory variables**	**Odds ratios**	**[95% confidence interval]**	**Coefficients**	**'t'**
Health rating	0	0.000009-0.0005	-9.676	-9.537*
Environment rating	26.76	12.43–57.60	3.287	8.404*
Residence	6.969	4.93–9.84	1.942	11.020*
Income	1.001	1.00-1.00	0.0005	13.946*
Education	2.315	1.80–2.97	0.84	6.600*
Age	1.148	1.09–1.20	0.138	5.751*
Age squared	0.999	0.99–1.00	-0.0008	-3.401*
Race	0.787	0.59–1.04	-0.239	-1.691
Household size	0.891	0.86–0.93	-0.115	-5.519*
Occupation	0.733	0.54–0.99	-0.311	-1.971
Employment status	0.518	0.38–0.69	-0.657	-4.308*
Smoking	1.633	1.29–2.07	0.49	4.052*
Alcohol use	0.617	0.45–0.84	-0.483	-3.033*
Contraceptives use	0.372	0.26–0.52	-0.988	-5.700*
Marital status	1.841	1.49–2.27	0.611	5.765*
Constant	-	-	-4.385	-8.755
Sample size	3489			
χ^2^(15)	1438.62			
Prob > χ^2^	0			
Pseudo-R^2^	0.3379			
Log likelihood	-1409.7041			

Note: * Indicates the coefficients are statistically significant at 95% confidence level, based on a two-tailed test. On the basis of chi-square test of the log-likelihood ratio, the joint effects of estimated logistic model are statistically significant at the 0.1% level.

t_INCOME _= (β_INCOME_)/(SE_INCOME_) = (0.0004727)/(0.0000339) = 13.946

The decision rule is: reject the null hypothesis (H_0_: β = 0) if the calculated t-value, t_k_, is greater than the critical t-value, t_c_, as long as the sign of t_k _is the same as the sign of the coefficient implied in the alternative hypothesis (H_A_: β≠0). Otherwise, accept the null hypothesis (H_0_) that the estimated regression coefficient in question is not significantly different from zero. In the above example, the coefficient of income is statistically significant at 95% level of confidence, based on a two-sided test, since the computed t-value (13.946) is greater than the critical t-value (1.960).

The coefficient (β) of the estimated binary logit model measures the impact of a one-unit change in an explanatory variable (R_i_) on the log of odds of a health insurance policy ownership, holding other explanatory variables constant. The coefficients for environment rating, residence, income, education, age, smoking and marital status are statistically significant at 95% level of confidence, and have positive signs. The latter result implies that an increase in any of these variables spontaneously impacts positively on the log of odds of health insurance policy ownership, holding other factors constant. Contrastingly, the coefficients for household size, alcohol, contraceptive use, age-squared, occupation, employment and health rating are statistically significant, and have got a negative effect on the log of odds of health insurance policy ownership.

## Discussion

### Health rating

Social scientists define health as a product of life expectancy (measured in years) and health-related quality of life (i.e. mobility, activities of daily living, social participation, pain, anxiety/depression, energy) [22,23]. The SANHIS data set [10] contained only self-evaluated categorical health status data. The respondents rated their current health status as either excellent, very good, good, fair or poor. The variable was re-coded into a dichotomous variable: 1 = excellent, very good, or good; and 0 if fair or poor.

An individual's stock of health determines the total amount of time he/she can spend producing commodities and money earnings [24]. Health status is an important determinant of both earnings and capacity for enjoying life. A decline in the death rate at working ages may improve earning prospects by extending the period during which earnings are received [25]. The coefficient for the health status variable took a negative sign, implying that the demand for health insurance was likely to be low among individuals who were in excellent, very good or good health. In the current study, 58.3% of the respondents in the sub-sample with a health insurance policy assessed their health status as either fair or poor. This may be a case of adverse selection [26], which results in insurance having the greatest appeal to individuals who are more likely to fall sick [9,27]. Adverse selection, depending on its extent, could jeopardize the economic viability of a health insurance scheme.

The adverse selection problem can be curbed in two main ways without compromising equity objectives, namely: (i) compulsory social health insurance (or a national health service) for a defined population through legislation – not an option for private health insurance which is voluntary by definition; and (ii) the government could step in and provide health insurance to all those at exceptionally high risk (e.g. the elderly and those with chronic diseases) and the poor who cannot afford the premiums. Commercial health insurance firms often curb adverse selection by introducing experience rating, i.e. linking insurance premium to the degree of assessed risk of falling sick (this action may have negative equity implications); and/or subjecting all those who apply for insurance cover to a thorough medical examination (this could potentially lead to cream-skimming, excluding all those with high risks of falling sick).

### Economic factors

There were three economic variables, namely, income (+), occupation (-) and employment (-), where (.) is the hypothesized sign of the coefficients. The coefficients of the three variables were statistically significant and had the expected signs. High incomes, white-collar occupations and being gainfully employed are significant predictors of health insurance ownership. The proportion of people with health insurance rises considerably as one moves up the household income distribution ladder, with the coverage going from 6.3% of those in the income range 1–950 Rand to over 90.7% among those earning 7600 Rand and above per month. The trend is similar to that reported by Harmon and Nolan [16] in Ireland and Propper [17] in England and Wales. This implies that any macroeconomic interventions aimed at decreasing involuntary unemployment and boosting disposable incomes among households will spontaneously increase the probability of health insurance ownership. Thus, the post-apartheid South African government's economic programmes of black empowerment, small-scale micro financing programmes and land (and other assets) redistribution programmes are likely to increase the number of households with the ability to purchase health insurance policies.

### Demographic factors

The demographic factors include: age (+), age squared (-) and household size (-), where (.) is the postulated sign of the coefficient. The coefficients for age and age squared were statistically significant at the 5% level. Economic theory predicts that as individuals advance in age, their inherited health stock depreciates at an increasing rate (a manifestation of the biological process of ageing) and they tend to increase investments in health (including health insurance) in an attempt to decrease the rate of depreciation. This is consistent with Grossman's findings [24] that because the health stock depreciation rate rises with age, it is not unlikely that unhealthy (old) people will make larger gross investments in health than healthy (young) people.

The coefficient for household size variable had a statistically significant negative effect on the likelihood of health insurance policy ownership. This finding is intuitively sensible since any increase in the household size, while holding the income constant, reduces the per capita income.

### Social factors

The social factors include: education (+) and marital status (+). The coefficient for education was statistically significant, and had the expected positive sign. Respondents with at least a matriculation (secondary) level of education were two times more likely to be in possession of a health insurance policy than those with a lower level of education. This could be attributed to a positive relationship between a person's: (i) educational level and propensity to acquire skills; (ii) stock of knowledge and his/her market and non-market productivity [24] and earnings; and (iii) education and knowledge about the advantage of making regular small insurance payments to avoid the risk of catastrophic medical expenditures [28].

Marital status had a statistically significant positive effect on health insurance ownership. Married persons are more likely to have insurance cover than those who are single, separated or divorced. This finding is consistent with the result obtained by Harmon and Nolan [16], Rhine et al. [12], Trujillo [14] and Liu and Chen [28]. Married couples may have a higher demand for health insurance due to: (i) the need to protect their children [16]; (ii) higher combined income; and (iii) being more averse to the risk of catastrophic health expenditures than those who are single, separated or divorced.

### Spatial and environmental factors

The spatial and environmental factors – residence (+) and environment rating (+) – had a statistically significant effect on health insurance ownership. The respondents living in formal urban settlements or rural white-owned farms had a seven times higher odds of owning a health insurance policy than those living in informal urban settlements or former rural homelands. This could partly be a reflection of the economic well being of the former group.

The respondents who felt that the environment they lived in was good, very good or excellent were twenty-seven times more likely to have a health insurance cover than those who lived in fair or poor environments. This phenomenon may be a reflection of a better socio-economic status of those living in relatively affluent, formal (and cleaner) residential areas *vis-à-vis *informal settlements (which are relatively deprived of all kinds of social amenities).

### Behavioural factors

The behavioural factors included in the analysis were: contraceptive use (-), alcohol use (-) and smoking (+). The three had a statistically significant effect on the demand for health insurance. The coefficient for contraceptives assumed a negative sign. This implies that the use of contraceptives may not necessarily be linked with individuals' attitudes toward risk.

The coefficient for alcohol use also took a negative sign, which implies that those people who drank alcohol were less likely to purchase health insurance. On the contrary, the parameter for smoking assumed a positive sign, meaning that being a cigarette smoker, the probability of a person demanding health insurance coverage increases. In the context of the health insurance market, the latter finding could be a source of concern if it were a signal for the presence of moral hazard. Moral hazard is a potential cost of insurance in which the presence of insurance increases the tendency for losses to occur through careless, irresponsible or perhaps illegal behaviour [26].

For example, the fully insured individuals may embark on risky behaviours, such as smoking, and by so doing expose themselves to a high risk of developing various forms of cancer (throat cancer, lung cancer, etc.). Insurers attempt to control moral hazard by careful underwriting of applicants for insurance and by various policy provisions, such as deductibles (which requires an individual to pay for a certain amount of health care received before the insurance comes into effect) and co-insurance/co-payment (which requires the insured person to pay a certain percentage of eligible medical expenses in excess of the deductibles, with the insurer paying the remainder) [15,29].

This study used consumption of contraceptives, alcoholic drinks and cigarettes as proxies for consumers' attitudes toward health risks. The three may not be ideal proxies for risk attitudes; however, there were no better alternatives in the data set. If this were a study primarily designed to analyse the demand for health insurance, it would have been preferable to either proxy risk attitudes using a qualitative scale variable ranging from 1 (extremely risk-averse) to 10 (risk-lover) [11], directly estimate the revealed risk-aversion using experimental data [29], or ask the respondent to report whether he/she would consider paying for private health insurance at the point of demand [30].

### Limitation of the study

The main weakness of the current study is that since the data set upon which the analysis was based was gathered for a different purpose (i.e. it was not dedicated to health insurance), it did not contain insurance-specific attributes, e.g. premiums, co-payments, deductibles, benefits covered and the quality of care in the health facilities where the insured sought care. Thus, we had a situation where important explanatory variables were left out of the estimated regression equation 2 (in the *Appendix*), leading to specification bias or omitted variable bias [21]. The omission of a relevant independent variable can change the estimated coefficient away from the true value of the population coefficient.

### Further research

In sub-Saharan Africa, there is need for studies on the following:

• The determinants of private and social health insurance policy ownership, which include both health insurance programme attributes (e.g. premiums, co-payments, deductibles) and household socio-economic characteristics (including attitudes toward risk).

• The willingness and ability to pay for various forms of health insurance, including voluntary and non-voluntary insurance schemes [31].

• The economic viability of various forms of health insurance, e.g. social health insurance and community-based prepaid schemes.

• Design of innovative health insurance schemes; for example, within farmers' cooperative societies [36], savings and credit societies, agricultural estates, women/men developmental groups [37], civil service, etc.

• Whether the expansion of private health insurance under the current health care delivery system would yield significant public sector cost savings, and improved targeting of subsidies for the poor and preventive services [32].

• Optimal ways of curbing health insurance problems of moral hazard and adverse selection.

• Benefit-incidence analysis of alternative health insurance arrangements.

## Conclusion

The environment rating, residence, income, education, age, smoking and marital status variables were all found to have a statistically significant (at 95% confidence level) positive relationship with ownership of health insurance schemes. Contrastingly, the other covariates, namely: health rating, age squared, household size, occupation, employment, alcohol use and contraceptive use had a significantly negative relationship with health insurance ownership.

There are a number of policy implications of this study:

• High incomes, white-collar occupations and being gainfully employed are significant predictors of health insurance ownership. Thus, economic development (or poverty reduction) programmes geared at: (i) improving incomes of the vulnerable segments of the South African population; (ii) reducing involuntary unemployment; and (iii) creating white-collar job opportunities will empower South African women to reach a higher standard of living and in doing so boost their economic access to health insurance policies and the relevant health services.

• Policies aimed at ensuring that the majority of South Africans attain a matriculation (i.e. secondary) education level will increase by almost two-fold, the probability of acquiring health insurance.

• The self-assessed health status was found to have a statistically negative effect on the demand for health insurance. This implies existence of adverse selection. However, this problem can be reduced through compulsory social health insurance [33]; or through state insurance for high-risk groups, particularly the poor. Since 53% of the South African population lives below the income poverty line of US$2 per day [34], implementation of the social health insurance programme [7] would increase access to basic health services by poor.

## Competing interests

The author(s) declare that they have no competing interests.

## Authors' contributions

JMK recoded the raw data and participated in the development of the conceptual framework, analysis and drafting of sections of the document. LGS, BN, GMM, RC and TM participated in the development of the conceptual framework and drafting of sections of the document. All authors read and approved the final manuscript.

## Appendix: Empirical model

A binary logit model was used in the analysis of individual household's choice between having no health insurance and having health insurance. We assumed that the expected utility associated with each health insurance option is a function of a vector of its attributes (X_i_) and a vector of a household's socioeconomic characteristics (R_i_), plus a stochastic error term (ε). The latter component captures errors in model specification (e.g. omission of relevant variables) and errors in data measurement.

Algebraically, a household's decision process can be expressed as:

*EU*_*ij *_= *g*(*X*_*ij*_, *R*_*i*_) + *ε *.................................... *(1)*;

where: EU_ij _is the utility that i^th ^household expects to derive from choosing j^th ^health insurance option; j = 1 if a household has health insurance; j = 2 if a household has no health insurance; and X, R and ∈ are as defined above.

The basic assumption is that the i^th ^household opts for 'health insurance' if EU_i1 _> EU_i2_, prefers 'no health insurance' if EU_i1 _< EU_i2, _and is indifferent between the two options if EU_i1 _= EU_i2_. Thus, the probability that i^th ^household prefers to have health insurance is: P_i1 _= P(EU_i1 _> EU_i2_). And, conversely, the probability that i^th ^household prefers not to have health insurance is: P_i2 _= P(EU_i1 _< EU_i2_).

To determine the probability of health insurance ownership, the following model was estimated:

*P*_*ij *_= (*α *+ *β*_1 _*HEALTH_RATING + β*_2 _*ENVIRONMENT_RATING + β*_3 _*RESIDENCE + β*_4 _*INCOME + β*_5_*EDUCATION + β*_6 _*AGE + β*_7 _*AGE_SQUARED + β*_8 _*RACE + β*_9 _*HOUSEHOLD_SIZE + β*_10 _*OCCUPATION + β*_11 _*EMPLOYMENT + β*_12 _*SMOKING + β*_13 _*ALCOHOL_USE + β*_14 _*CONTRACEPTIVES_USE + β*_15 _*MARITAL_STATUS + ε*_*i*_........................ *(2)*

where: P_ij _= 1 if individual 'i' owns insurance (j = 1) and equals zero otherwise (j = 0); (*α*) is the intercept term; (β's) are the estimated coefficients; and *ε*_i _is the stochastic error term. The explanatory variables included in the model are defined in Table 1. Because of the limitations associated with linear probability model, the logit version of equation 2 was estimated, using maximum Likelihood Method.

## Pre-publication history

The pre-publication history for this paper can be accessed here:


